# Supplementary material for: Evolution of high-molecular-mass hyaluronic acid is associated with subterranean lifestyle
Source: Nat Commun. 2023 Dec 5;14:8054. doi: 10.1038/s41467-023-43623-2 (PMC10698142; doi:10.1038/s41467-023-43623-2)
Supplement: Supplementary file 1 — Supplementary Information [file 41467_2023_43623_MOESM1_ESM.pdf]

Supplementary Information for

Evolution of High-Molecular-Mass Hyaluronic Acid is Associated with Subterranean Lifestyle

Yang Zhao[1, 2, #], Zhizhong Zheng[1, #], Zhihui Zhang[1], Yandong Xu[2], Eric Hillpot[1], Yifei S. Lin[1], Frances T. Zakusilo[1], J. Yuyang Lu[1], Julia Ablaeva[1], Seyed Ali Biashad[1], Richard A. Miller[3], Eviatar Nevo[4], Andrei Seluanov[1, *], Vera Gorbunova[1, *]

1 Departments of Biology and Medicine, University of Rochester, Rochester, NY 14627, USA

2 Department of Physiology and Department of Hepatobiliary and Pancreatic Surgery of the First Affiliated Hospital, Zhejiang University School of Medicine, Hangzhou 301158, China

3 Department of Pathology, University of Michigan Medical School, Ann Arbor, Michigan 48109, USA

4 Institute of Evolution, University of Haifa, Haifa 3498838, Israel

# These authors contributed equally to this work

* Corresponding authors:

Vera Gorbunova

Email: vera.gorbunova@rochester.edu

Andrei Seluanov

Email: andrei.seluanov@rochester.edu

This PDF file includes:

Supplementary Figures S1-7

Supplementary Figure legends

Other supplementary materials for this manuscript include:

Supplementary Data 1-3

Figure S1

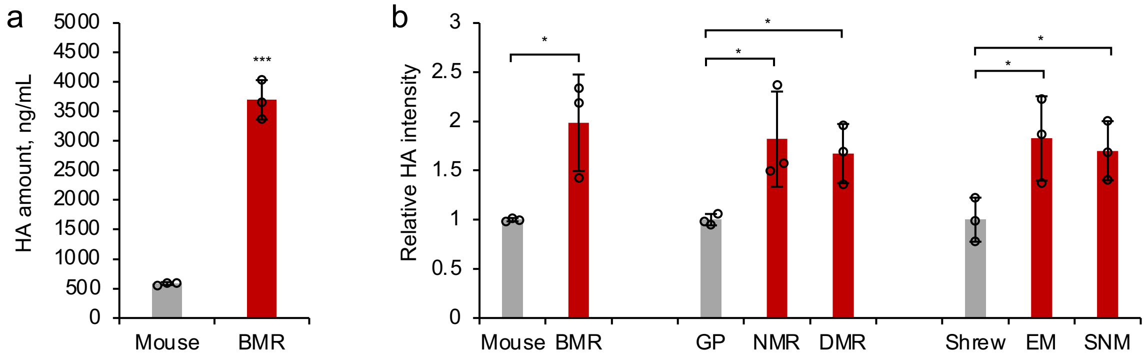

Fig. S1. Quantification of HA in conditioned media. (a) ELISA quantification of HA from media of BMR and mouse cells. Data are mean ± SD of biological replicates. *** $P < 0.001$ by unpaired two-sided t-test. (b) Quantification of HA intensity from pulsed-field gel. HA purified from media of different cell lines was subjected to pulsed-field electrophoresis and stained with Stains-All. The relative HA intensity was quantified using ImageJ. Data are mean ± SD of gels from three independent experiments. * $P < 0.05$ by unpaired two-sided t-test. Source data are provided as a Source Data file.

Figure S2

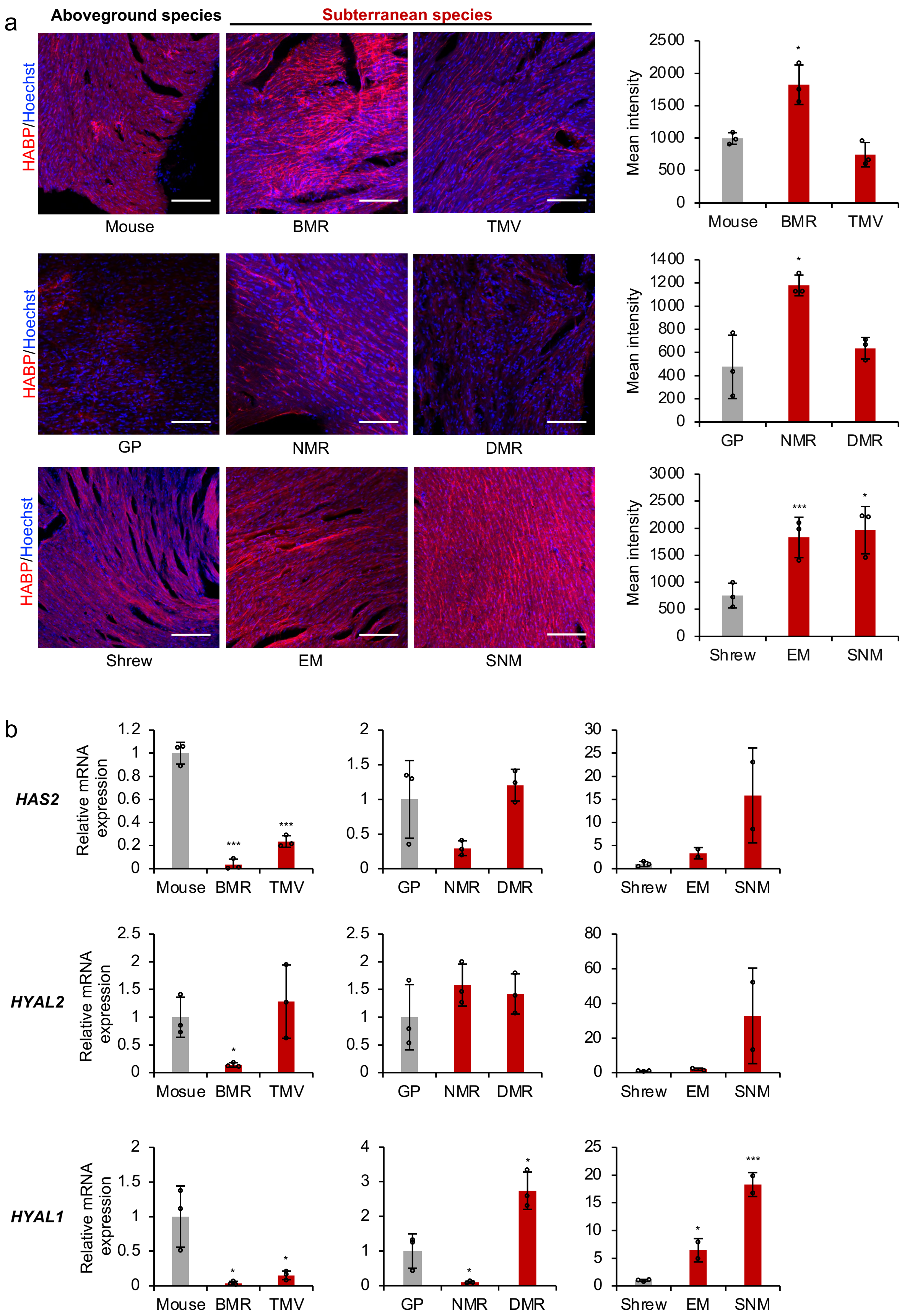
a
Aboveground species
Subterranean species
HABP/Hoechst
Mouse
BMR
TMV
GP
NMR
DMR
Shrew
EM
SNM
Mean intensity
b
HAS2
HYAL2
HYAL1
Relative mRNA expression
Mosue

Fig. S2. Levels of HA in the heart of subterranean species. (a) Cryosections of heart tissues from different species were stained with hyaluronan binding protein (HABP, red) and Hoechst (blue). Experiments were repeated three times. Representative Immunofluorescence images are shown at 20 × magnification (left). Scale bar, 50 μm. Quantification of HABP fluorescence (right). Data are mean ± SD of three independent areas. *P*-value indicates the difference between subterranean and the corresponding aboveground species. * $P < 0.05$, *** $P < 0.001$ by unpaired two-sided t-test. (b) RT-qPCR showing expression levels of *HAS2* and *HYAL2* and *HYAL1* genes in the heart comparing subterranean species with their aboveground controls. Experiments were performed using three biological replicates, except EM and SNM, which had two biological replicates. *P*-value indicates the difference between subterranean and the corresponding aboveground species. Data are mean ± SD. * $P < 0.05$, *** $P < 0.001$ by unpaired two-sided t-test. Source data are provided as a Source Data file.

Figure S3

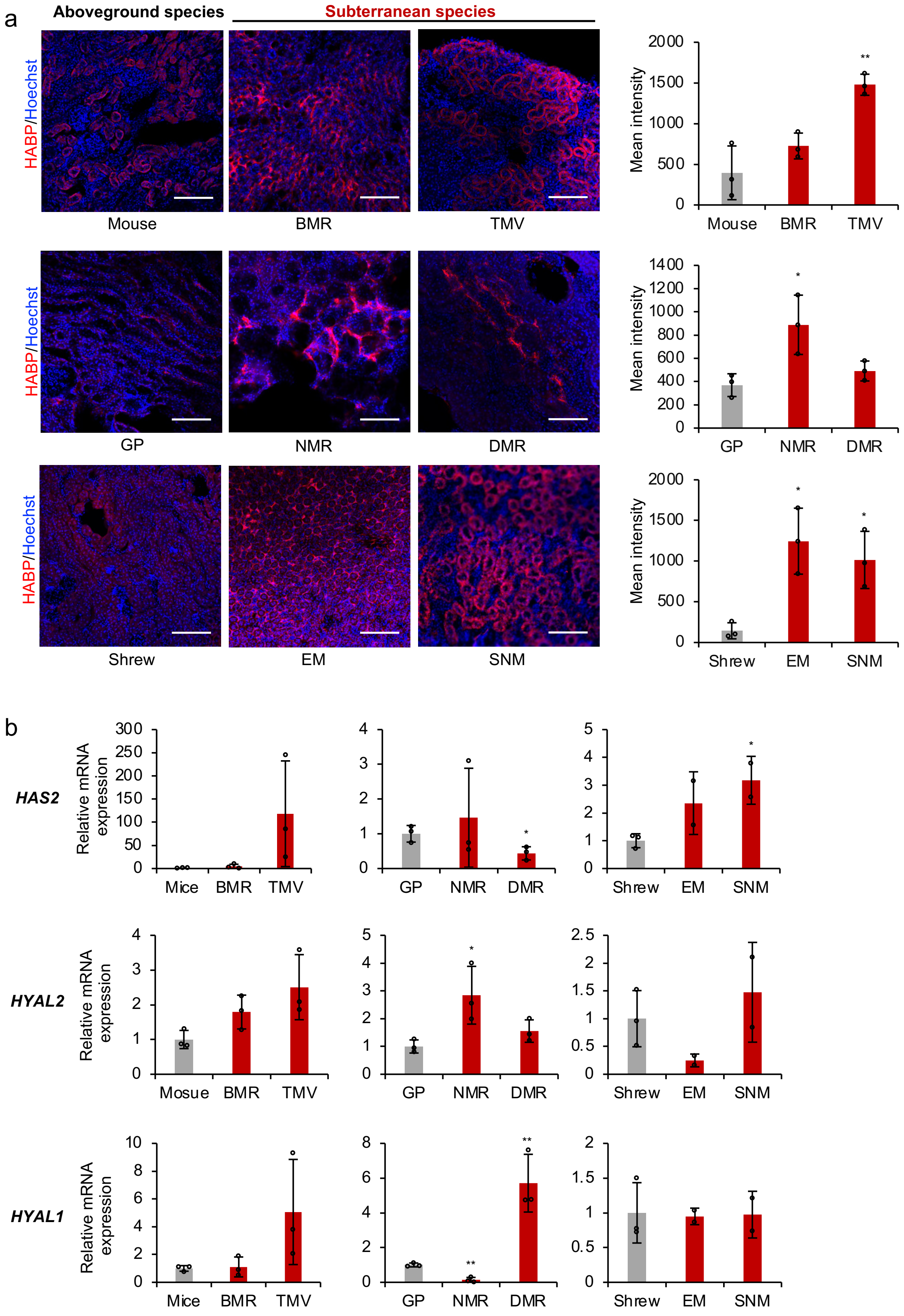
a
Aboveground species
Subterranean species
HABP/Hoechst
Mouse
BMR
TMV
GP
NMR
DMR
Shrew
EM
SNM
Mean intensity
b
HAS2
HYAL2
HYAL1
Relative mRNA expression
Mice
Mosue

Fig. S3. Levels of HA in the kidney of subterranean species. (a) Cryosections of kidney tissues from different species were stained with hyaluronan binding protein (HABP, red) and Hoechst (blue). Experiments were repeated three times. Representative immunofluorescence images are shown at 20 × magnification (left). Scale bar, 50 μm. Quantification of HABP fluorescence (right). Data are mean ± SD of three independent areas. *P*-value indicates the difference between subterranean and the corresponding aboveground species. * $P < 0.05$, *** $P < 0.001$ by unpaired two-sided t-test. (b) RT-qPCR showing expression levels of *HAS2* and *HYAL2* and *HYAL1* genes in the heart comparing subterranean species with their aboveground controls. Experiments were performed using three biological replicates, except EM and SNM, which had two biological replicates. *P*-value indicates the difference between subterranean and the corresponding aboveground species. Data are mean ± SD. * $P < 0.05$, *** $P < 0.001$ by unpaired two-sided t-test. Source data are provided as a Source Data file.

Figure S4

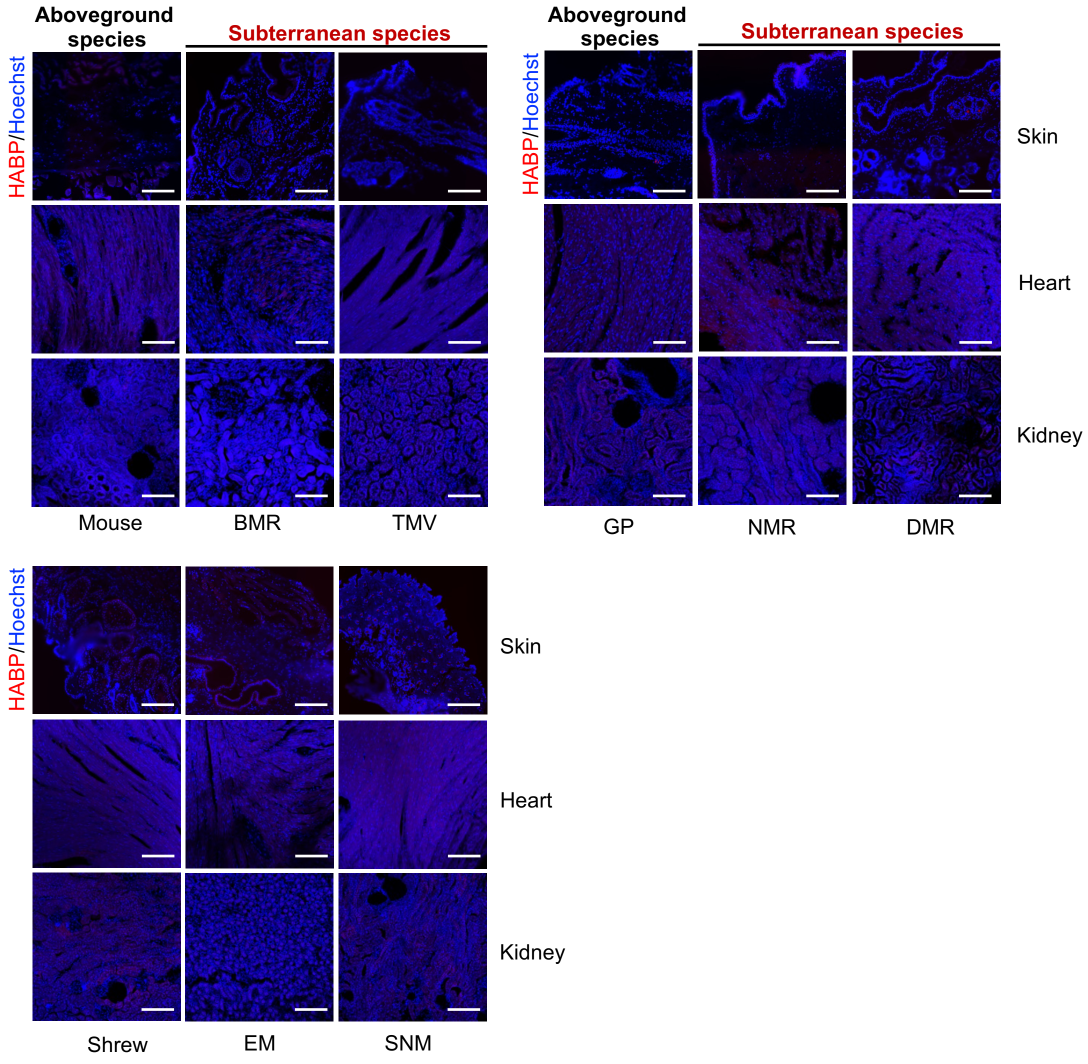

Fig. S4. Negative controls for tissue HABP staining. Cryosections of skin, heart, and kidney were treated with HAase overnight before staining with HABP. The negative signal for HABP channel indicates the specificity of HABP staining for HA. Scale bar, 50 μm.

Figure S5

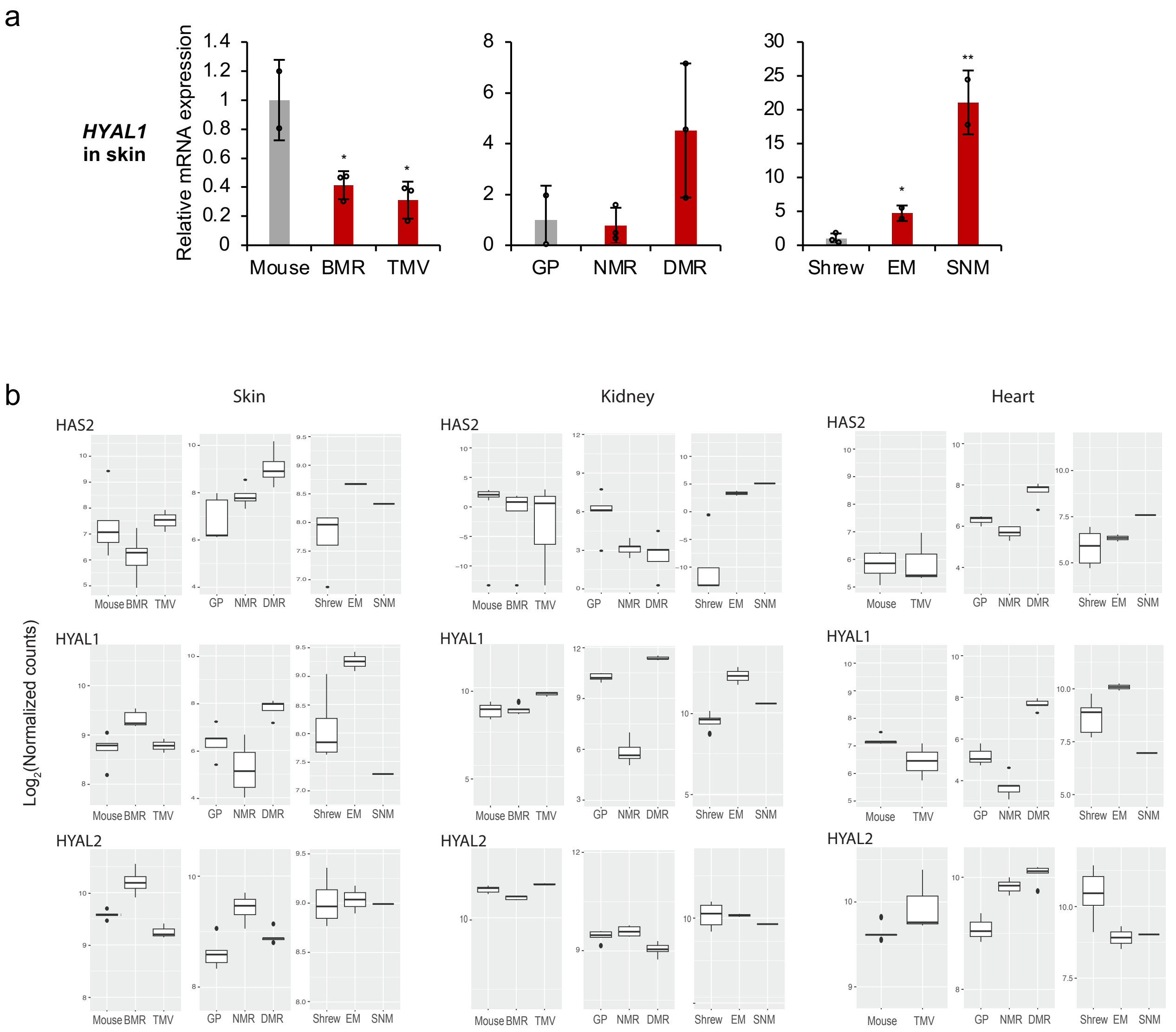

Fig. S5. Expression of HAS2, HYAL2, and HYAL1 in the tissues of subterranean species. (a) RT-qPCR was performed using three biological replicates, except Mouse, GP, EM and SNM, which had two biological replicates. Data are mean ± SD. * $P < 0.05$ by unpaired two-sided t-test. (b) Boxplots showing the expression of HAS2, HAYL1, and HYAL2 in skin, kidney and heart of different species determined by RNAseq. Log2 transformed normalized counts were shown. The boxplots display the median, the 1st, and 3rd quartiles; the whiskers show a 1.5× interquartile range. Data points outside the whiskers are outliers. Skin: Mouse n = 5, BMR n = 5, TMV n = 3, GP n = 5, NMR n = 4, DMR n = 5, Shrew n = 4, EM n = 2, and SNM n = 1; Kidney: Mouse n = 10, BMR n = 5, TMV n = 3, GP n = 5, NMR n = 5, DMR n = 5, Shrew n = 8, EM n = 2, and SNM n = 1; Heart: Mouse n = 5, TMV n = 3, GP n = 3, NMR n = 5, DMR n = 5, Shrew n = 10, EM n = 2, and SNM n = 1. Source data are provided as a Source Data file.

Figure S6

a

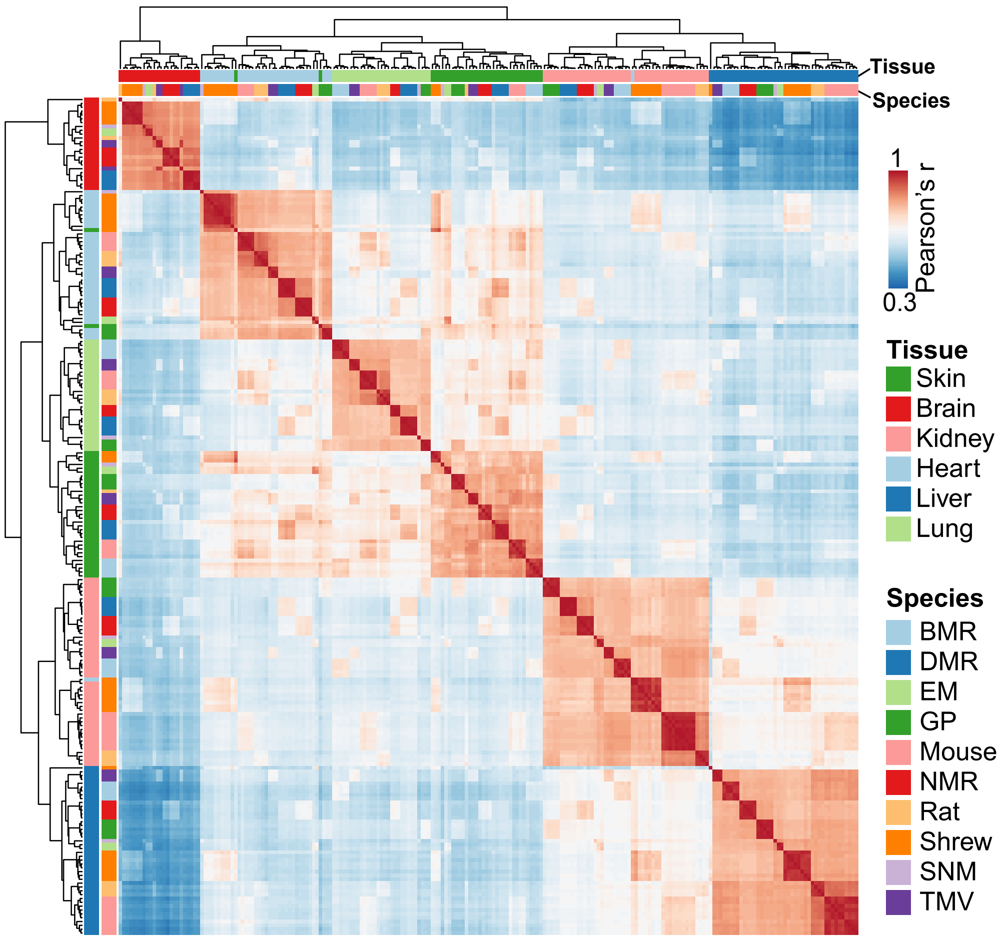
Tissue
Species
1
Pearson's r
0.3
Tissue
Skin
Brain
Kidney
Heart
Liver
Lung
Species
BMR
DMR
EM
GP
Mouse
NMR
Rat
Shrew
SNM
TMV

b

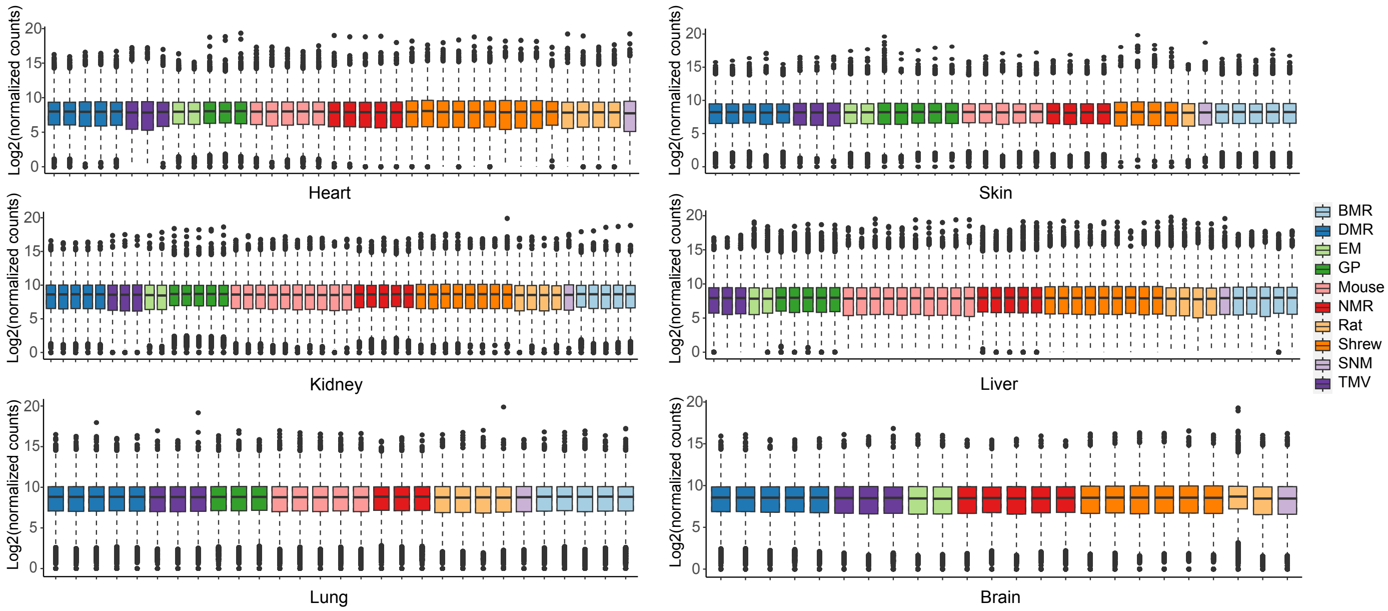
Log2(normalized counts)
20
15
10
5
0
Heart
Skin
Kidney
Liver
Lung
Brain
BMR
DMR
EM
GP
Mouse
NMR
Rat
Shrew
SNM
TMV

Fig. S6. Quality controls for RNA sequencing (RNAseq). (a) Heatmap showing the Pearson correlation coefficient among all samples. Samples are mainly clustered by tissues. (b) Boxplot of gene expression (in log scale) of individual samples for each tissue after filtering out low-expressed genes and normalized by DESeq2 package. The boxplots display the median, the 1st, and 3rd quartiles; the whiskers show a 1.5× interquartile range. Data points outside the whiskers are outliers. Same number of genes were shown for each species: Heart n = 13,089, Kidney n = 13,323, Lung n = 13,395, Skin n = 13,211, Liver n = 13,023, and Brain n = 13,452.

Figure S7

a

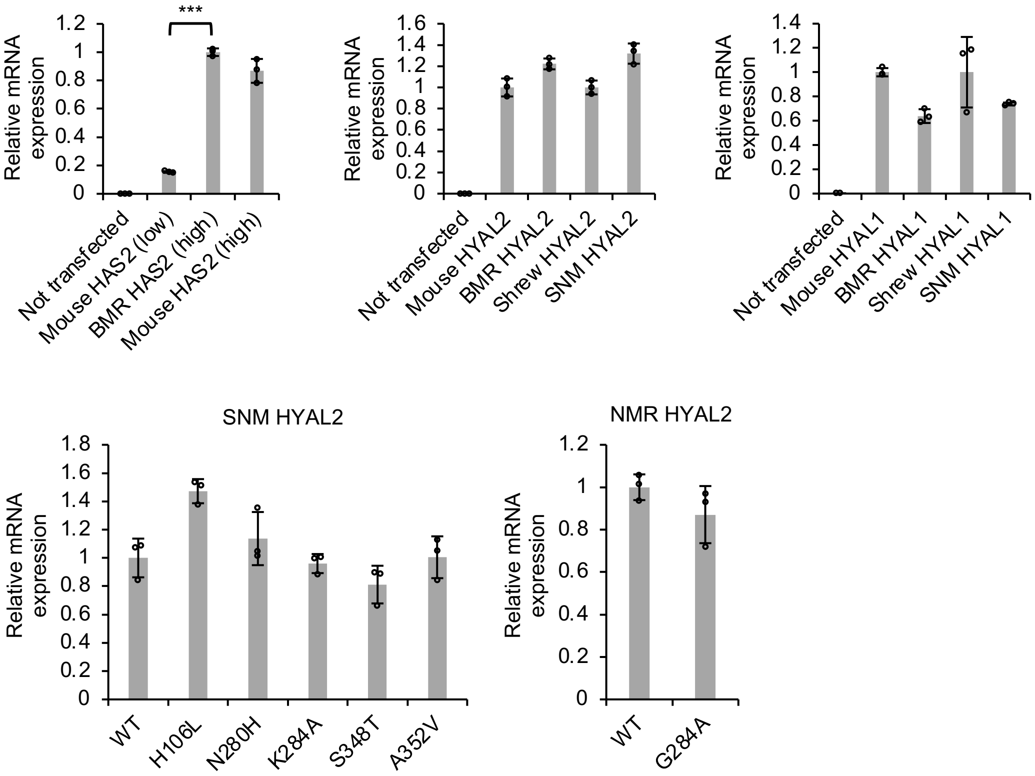
***
Relative mRNA expression
1.2
1
0.8
0.6
0.4
0.2
0
Not transfected
Mouse HAS2 (low)
BMR HAS2 (high)
Mouse HAS2 (high)
1.6
1.4
Not transfected
Mouse HYAL2
BMR HYAL2
Shrew HYAL2
SNM HYAL2
Not transfected
Mouse HYAL1
BMR HYAL1
Shrew HYAL1
SNM HYAL1
SNM HYAL2
1.8
WT
H106L
N280H
K284A
S348T
A352V
NMR HYAL2
G284A

b

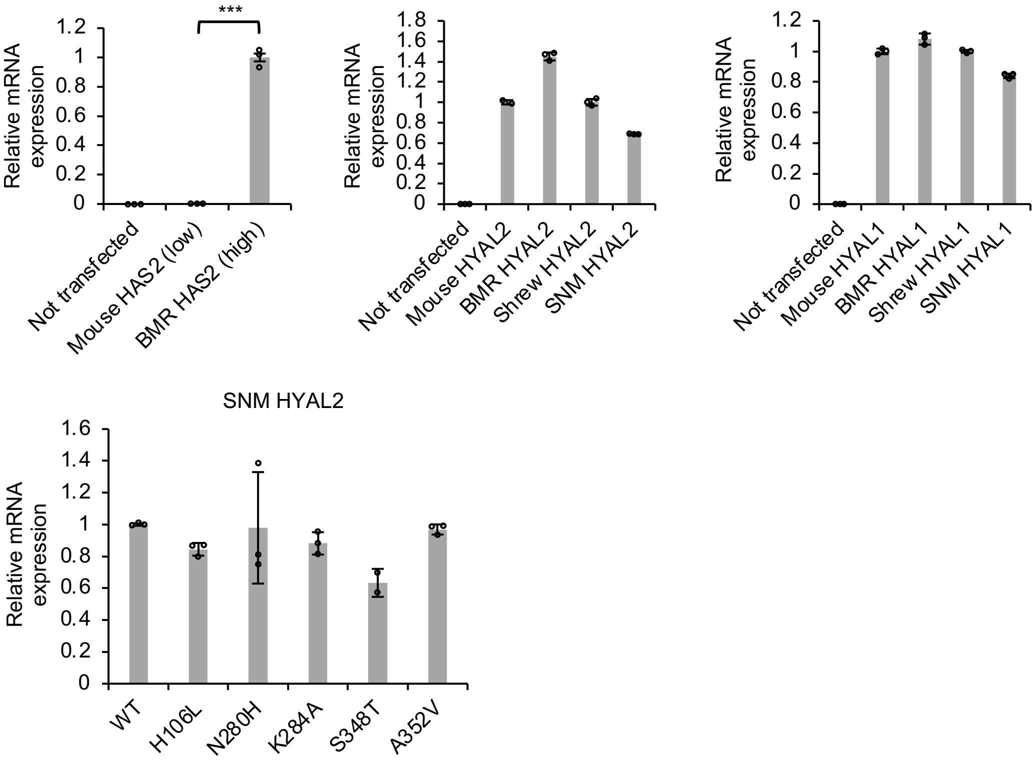
***
Relative mRNA expression
1.2
1
0.8
0.6
0.4
0.2
0
Not transfected
Mouse HAS2 (low)
BMR HAS2 (high)
1.8
1.6
1.4
Not transfected
Mouse HYAL2
BMR HYAL2
Shrew HYAL2
SNM HYAL2
Not transfected
Mouse HYAL1
BMR HYAL1
Shrew HYAL1
SNM HYAL1
SNM HYAL2
WT
H106L
N280H
K284A
S348T
A352V

Fig. S7. Expression levels of transfected genes. HeLa cells (a) and 293T cells (b) were transfected with low amount of mouse HAS2 or high amount of BMR HAS2 to mimic the different endogenous expression in skin fibroblasts. Other genes including HYAL1 and HYAL2 from mouse, BMR, shrew, SNM, as well as mutant HYAL2 of SNM and NMR were transfected equally. RT-qPCR was performed to determine expression levels of HAS2, HYAL2, and HYAL1 in transfected cells. Data are mean ± SD of three technical replicates. *** $P < 0.001$ by unpaired two-sided t-test. Source data are provided as a Source Data file.

Figure S8

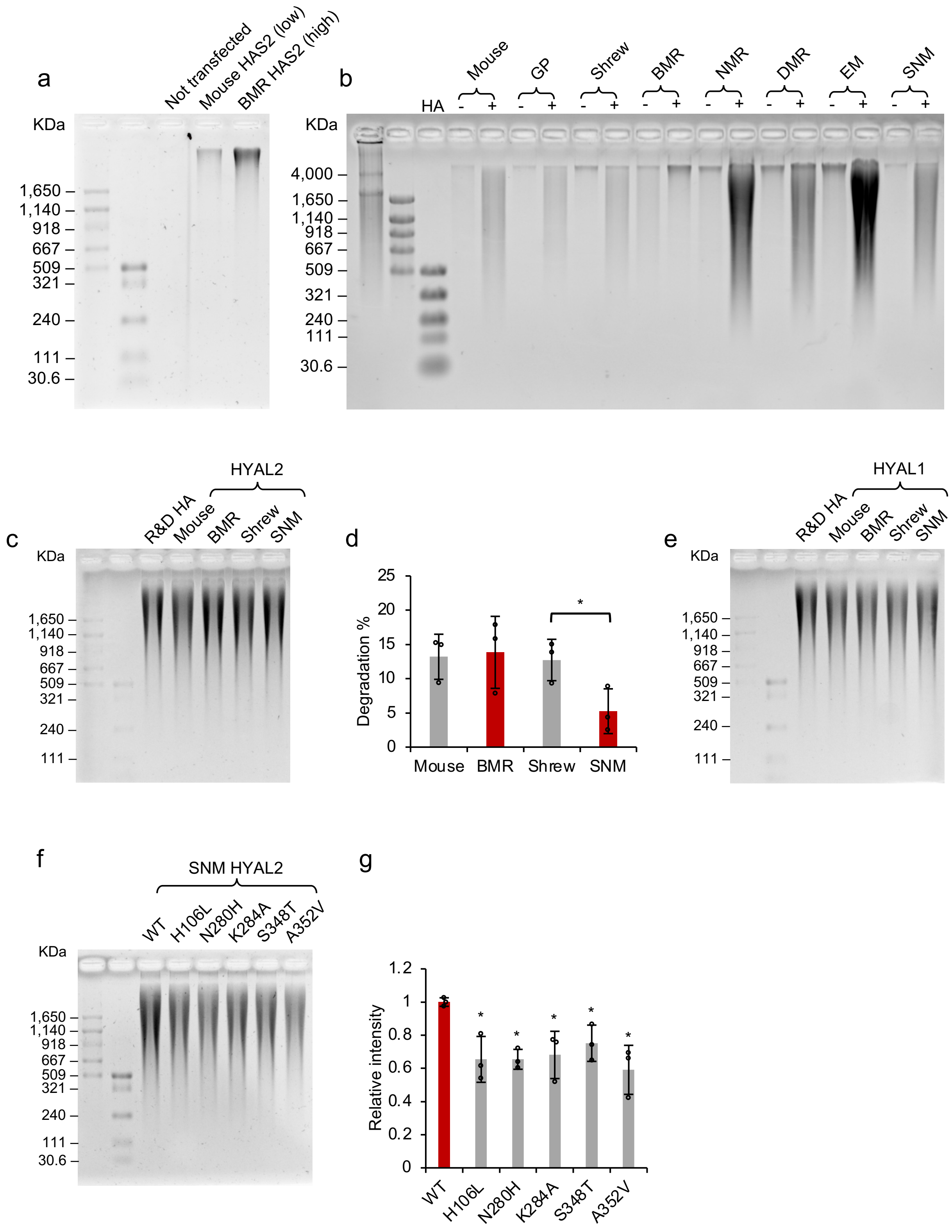

a
Not transfected
Mouse HAS2 (low)
BMR HAS2 (high)
KDa
1,650
1,140
918
667
509
321
240
111
30.6
b
HA
Mouse
GP
Shrew
BMR
NMR
DMR
EM
SNM
- +
4,000
c
HYAL2
R&D HA
d
Degradation %
*
e
HYAL1
f
SNM HYAL2
WT
H106L
N280H
K284A
S348T
A352V
g
Relative intensity

Fig. S8. Effects of HA-related enzymes on HA synthesis and degradation in subterranean species. (a) 293T cells were transfected with low amount of mouse HAS2 or high amount of BMR HAS2 (14-fold higher) plasmids. Two days after transfection, HA was purified and run on an agarose gel. (b) Media containing 20 μg/mL commercial HMM-HA was incubated with growing fibroblasts from each species. Two days after incubation, HA was purified from the media and run on an agarose gel. For each cell line, a sample without commercial HA was included to control for the endogenous HA. The amount of loaded HA was normalized to cell number of each species. (c-e) 293T cells were transfected with either HYAL2 (c) or HYAL1 (e) of mouse, BMR, shrew, or SNM. Transfected cells were cultured in media containing 20 μg/mL commercial HA. Two days after incubation, HA was purified and run on an agarose gel. (d) Quantification of HA gel in panel c. Degradation of HA by HYAL2 from different species was calculated based on the difference in intensity between R&D HA and the treated samples. HA intensities were quantified using ImageJ. (F) 293T cells were transfected with either wild-type (WT) SNM HYAL2 plasmid, or each of its five mutants. Cells were incubated with media containing 20 μg/mL commercial HA for two days, followed by HA extraction an electrophoresis. (g) Quantification of HA intensities after degradation by WT or mutant SNM HYAL2. For (d) and (g), data are mean ± SD of three technical replicates. * $P < 0.05$ by unpaired two-sided t-test. For (a), (b), and (e), experiments were performed three times with similar results. Source data are provided as a Source Data file.

Figure S9

a

```
Mouse 1   MHCERFLCVLRIIGTTLFGVSLLLGITAAYIVGYQFIQTDNYYFSFGLYGAFLASHLIIQSLFAFLEHRK 70
BMR   1   MHCERFLCILRIIGTTLFGVSLLLGITAAYIVGYQFIQTDNYYFSFGLYGAFLASHLIIQSLFAFLEHRK 70

Mouse 71  MKKSLETPIKLNKTVALCIAAYQEDPDYLRKCLQSVKRLTYPGIKVVMVIDGNSDDDLYMMDIFSEVMGR 140
BMR   71  MKKSLETPIKLNKTVALCIAAYQEDPDYLRKCLQSVKRLTYPGIKVVMVIDGNSDDDLYMMDIFSEVMGR 140

Mouse 141 DKSATYIWKNNFHEKGPGETEESHKESSQHVTQLVLSNKSICIMQKWGGKREVMYTAFRALGRSVDYVQV 210
BMR   141 DISATYIWKNNFHEKGPGETDESHKESSQHVTQLVLSNKSVCIMQKWGGKREVMYTAFRALGRSVDYVQV 210

Mouse 211 CDSDTMLDPASSVEMVKVLEEDPMVGGVGGDVQILNKYDSWISFLSSVRYWMAFNIERACQSYFGCVQCI 280
BMR   211 CDSDTMLDPASSVEMVKVLEEDPMVGGVGGDVQILNKYDSWISFLSSVRYWMAFNIERACQSYFGCVQCI 280

Mouse 281 SGPLGMYRNSLLHEFVEDWYNQEFMGNQCSFGDDRHLTNRVLSLGYATKYTARSKCLTETPIEYLRWLNQ 350
BMR   281 SGPLGMYRNSLLHEFVEDWYNQEFMGNQCSFGDDRHLTNRVLSLGYATKYTARSKCLTETPIEYLRWLNQ 350

Mouse 351 QTRWSKSYFREWLYNAMWFHKHHLWMTYEAVITGFFPFFLIATVIQLFYRGKIWNILLFLLTVQLVGLIK 420
BMR   351 QTRWSKSYFREWLYNAMWFHKHHLWMTYEAVITGFFPFFLIATVIQLFYRGKIWNILLFLLTVQLVGLIK 420

Mouse 421 SSFASCLRGNIVMVFMSLYSVLYMSSLLPAKMFAIATINKAGWGTSGRKTIVVNFIGLIPVSVWFTILLG 490
BMR   421 SSFASCLRGNIVMVFMSLYSVLYMSSLLPAKMFAIATINKAGWGTSGRKTIVVNFIGLIPVSVWFTILLG 490

Mouse 491 GVIFTIYKESKKPFSESKQTVLIVGTLIYACYWVMLLTLYVVLINKCGRRKKGQQYDMVLDV 552
BMR   491 GVIFTIYKESKKPFSESKQTVLIVGTLIYACYWVMLLTLYVVLINKCGRRKKGQQYDMVLDV 552
```

b

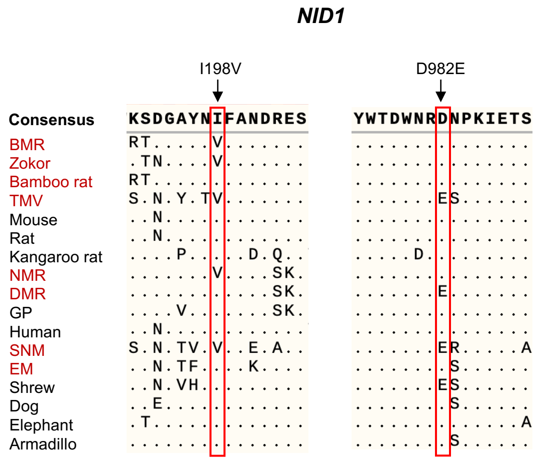

Fig. S9. Alignment of HAS2 and NID1 protein sequence. (a) Alignment of mouse and BMR HAS2 protein sequences. (b) Locations of two convergent mutation sites of NID1 protein across subterranean species are indicated with arrows. The names of subterranean species are labeled in red.

Figure S10

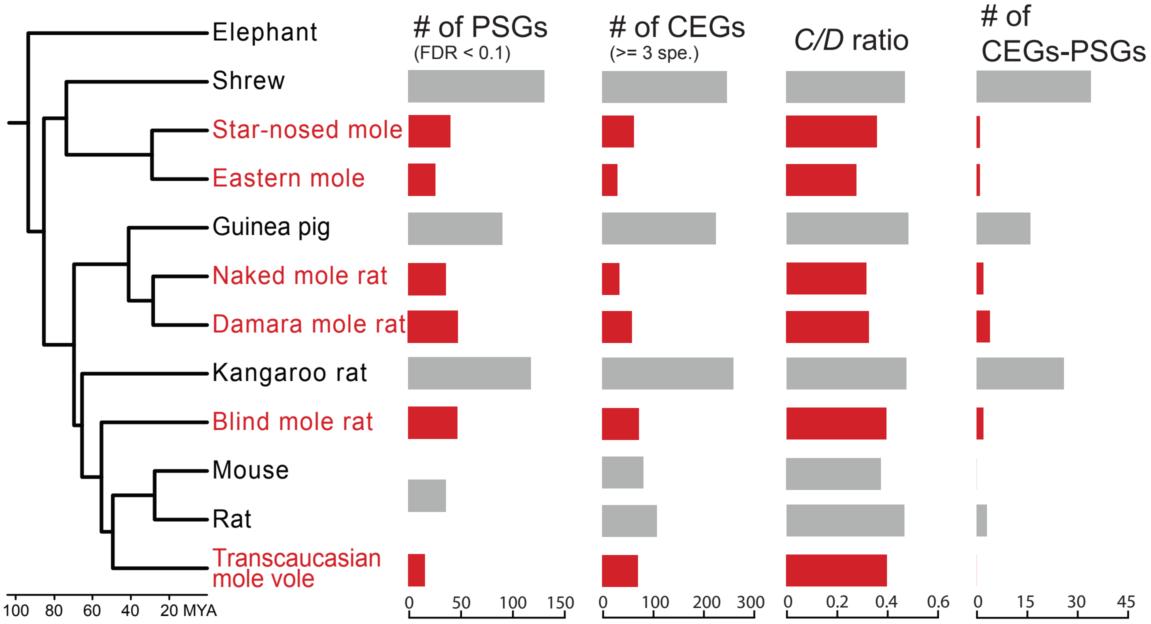

Fig. S10. Positively selected and convergently evolving genes in subterranean species. Phylogenetic tree of species without plateau zokor and hoary bamboo rat and their numbers of positively selected genes (PSGs), convergently evolving genes (CEGs), convergent-to-divergent site ratio (*C*/*D* ratio; >= 3 species) and genes with both positive selection sites and convergent sites (CEGs-PSGs). Error bars indicate the standard deviations of numbers of CEGs and *C*/*D* ratio resulting from different combinations of species. Subterranean species are shown in red.

Figure S11

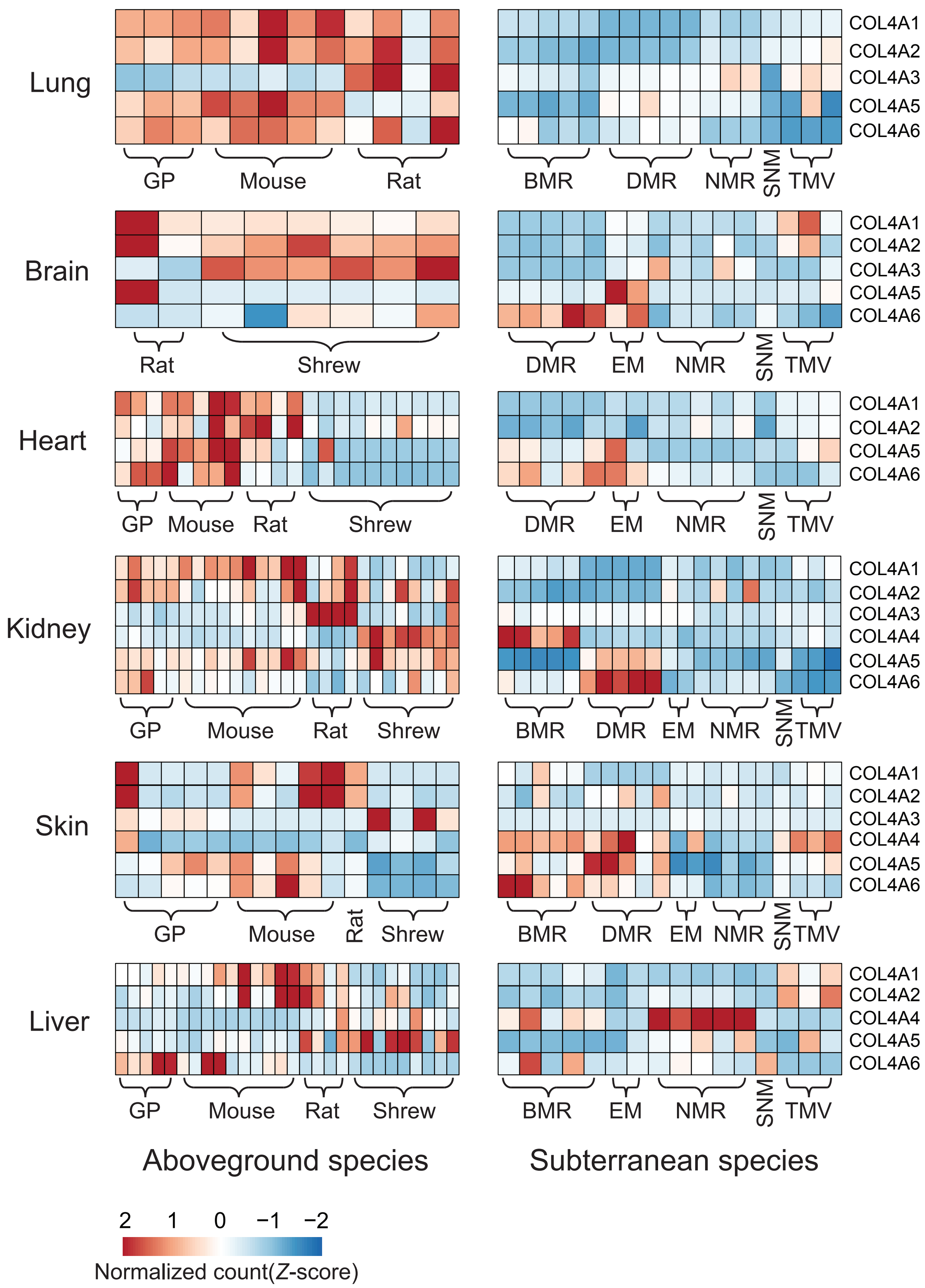
Lung
Brain
Heart
Kidney
Skin
Liver
COL4A1
COL4A2
COL4A3
COL4A4
COL4A5
COL4A6
GP
Mouse
Rat
Shrew
BMR
DMR
EM
NMR
SNM
TMV
Aboveground species
Subterranean species
2 1 0 −1 −2
Normalized count(Z-score)

Fig. S11. The heatmap showing expression of type IV collagen genes between subterranean and aboveground species. Heatmap showing expression of six genes including COL4A1, COL4A2, COL4A3, COL4A4, COL4A5 and COL4A6. COL4A1 and COL4A2 show consistently lower expression in most of the subterranean species in the lung, brain, heart, and kidney, but not in skin and liver.
