# Supplementary material for: Evolution of high-molecular-mass hyaluronic acid is associated with subterranean lifestyle
Source: Nat Commun. 2023 Dec 5;14:8054. doi: 10.1038/s41467-023-43623-2 (PMC10698142; doi:10.1038/s41467-023-43623-2)
Supplement: Supplementary file 3 — Description of Additional Supplementary Information [file 41467_2023_43623_MOESM3_ESM.pdf]

Description of Additional Supplementary Files

File Name: Supplementary Data 1
Description: Positively selected genes identified in different species and lineages using phylogeny including plateau zokor and bamboo rat.

File Name: Supplementary Data 2
Description: Positively selected genes identified in different species and lineages using phylogeny without plateau zokor and bamboo rat.

File Name: Supplementary Data 3
Description: ECM-related GO terms enriched by the higher expressed genes in subterranean skin and lung.
