# Supplementary material for: Evolution of high-molecular-mass hyaluronic acid is associated with subterranean lifestyle
Source: Nat Commun. 2023 Dec 5;14:8054. doi: 10.1038/s41467-023-43623-2 (PMC10698142; doi:10.1038/s41467-023-43623-2)
Supplement: Supplementary file 7 — Reporting Summary [file 41467_2023_43623_MOESM7_ESM.pdf]

nature portfolio

Corresponding author(s): Vera Gorbunova, Andrei Seluanov

Last updated by author(s): Oct 6, 2023

Reporting Summary

Nature Portfolio wishes to improve the reproducibility of the work that we publish. This form provides structure for consistency and transparency in reporting. For further information on Nature Portfolio policies, see our Editorial Policies and the Editorial Policy Checklist.

Statistics

For all statistical analyses, confirm that the following items are present in the figure legend, table legend, main text, or Methods section.

n/a	Confirmed	
☐	☒	The exact sample size (n) for each experimental group/condition, given as a discrete number and unit of measurement
☐	☒	A statement on whether measurements were taken from distinct samples or whether the same sample was measured repeatedly
☐	☒	The statistical test(s) used AND whether they are one- or two-sided<br>*Only common tests should be described solely by name; describe more complex techniques in the Methods section.*
☒	☐	A description of all covariates tested
☒	☐	A description of any assumptions or corrections, such as tests of normality and adjustment for multiple comparisons
☐	☒	A full description of the statistical parameters including central tendency (e.g. means) or other basic estimates (e.g. regression coefficient) AND variation (e.g. standard deviation) or associated estimates of uncertainty (e.g. confidence intervals)
☐	☒	For null hypothesis testing, the test statistic (e.g. F, t, r) with confidence intervals, effect sizes, degrees of freedom and P value noted<br>*Give P values as exact values whenever suitable.*
☐	☒	For Bayesian analysis, information on the choice of priors and Markov chain Monte Carlo settings
☒	☐	For hierarchical and complex designs, identification of the appropriate level for tests and full reporting of outcomes
☒	☐	Estimates of effect sizes (e.g. Cohen's d, Pearson's r), indicating how they were calculated

Our web collection on statistics for biologists contains articles on many of the points above.

Software and code

Policy information about availability of computer code

Data collection	Collection of qPCR data: ECFX Maestro"" Software by Bio-rad, version 1.0.<br>Microscope imaging: NIS-Elements by Nikon, version 4.50.
Data analysis	Excel was used to perform general statistical analyses (means, s.d., t-tests, etc).<br>For RNAseq data analyses, the following software and codes were used: Trim_Galore (version 0.6.6), DESeq2 (version 3.12).<br>For multiple alignment, the following softwares were used: Clustal Omega (release 1.2.2), Jalview (version 1.0).<br>ImageJ (version 1.50i) was used for immunofluorescence and HA quantification.

For manuscripts utilizing custom algorithms or software that are central to the research but not yet described in published literature, software must be made available to editors and reviewers. We strongly encourage code deposition in a community repository (e.g. GitHub). See the Nature Portfolio guidelines for submitting code & software for further information.

Data

Policy information about availability of data

All manuscripts must include a data availability statement. This statement should provide the following information, where applicable:

- Accession codes, unique identifiers, or web links for publicly available datasets
- A description of any restrictions on data availability
- For clinical datasets or third party data, please ensure that the statement adheres to our policy

Most data are included in the figures. RNAseq data used in this study are available in the Gene Expression Omnibus (GEO) under accession no. GSE181413 (https://www.ncbi.nlm.nih.gov/geo/query/acc.cgi?acc=GSE181413) and GSE190756 (https://www.ncbi.nlm.nih.gov/geo/query/acc.cgi?acc=GSE190756). The data generated in this study are provided in the Source Data file. The exact P values, if applicable, are included in the paper and in the Source Data.

Research involving human participants, their data, or biological material

Policy information about studies with human participants or human data. See also policy information about sex, gender (identity/presentation), and sexual orientation and race, ethnicity and racism.

Reporting on sex and gender	Not applicable
Reporting on race, ethnicity, or other socially relevant groupings	Not applicable
Population characteristics	Not applicable
Recruitment	Not applicable
Ethics oversight	Not applicable

Note that full information on the approval of the study protocol must also be provided in the manuscript.

Field-specific reporting

Please select the one below that is the best fit for your research. If you are not sure, read the appropriate sections before making your selection.

☐ Life sciences ☐ Behavioural & social sciences ☒ Ecological, evolutionary & environmental sciences

For a reference copy of the document with all sections, see nature.com/documents/nr-reporting-summary-flat.pdf

Ecological, evolutionary & environmental sciences study design

All studies must disclose on these points even when the disclosure is negative.

Study description	Fibroblasts and tissues of a group of subterranean mammal species were isolated to analyze the production of high-molecular-mass hyaluronic acid (HMM-HA). Electrophoresis, Carbazole assay, qPCR, immunofluorescence, RNA sequencing, and ELISA were used to quantify the production of HMM-HA and the expression of HA synthesis and degrading genes. For cells and tissues, experiments were performed using biological replicates as indicated in the figure legends. For HA electrophoresis, experiments were performed independently for three times with similar results.
Research sample	Fibroblasts and tissues of naked mole rats (NMR, Heterocephalus glaber), blind mole rats (BMR, Nannospalax galili), Damaraland mole rats (DMR, Fukomys damarensis), Transcaucasian mole vole (TMV, Ellobius lutescens), Eastern moles (EM, Scalopus aquaticus), star-nosed moles (SNM, Condylura cristata), and short-tailed shrews (shrew, Blarina brevicauda) were obtained. Young adults were selected based on their body weights. Sexes were randomly distributed.
Sampling strategy	The size of all samples are described in figure legends for all experiments. Sample sizes were selected empirically from previous experimental experience with similar assays, and/or from sizes generally employed in the field. For biological replicates, samples from three independent animals were used. No statistical test was used to predetermine sample size.
Data collection	Data were collected at the lab from cells or tissues using Electrophoresis, Carbazole assay, qPCR, immunofluorescence, RNA sequencing, and ELISA by the authors of this paper.
Timing and spatial scale	Data collection from the tissues and cells started from April 2021 to August 2023. All data were collected in the lab.
Data exclusions	No exclusion of data was made.
Reproducibility	All experimental data was reliably reproduced in multiple independent experiments as indicated in the figure legends.

Randomization	All tissues and primary fibroblasts were isolated from randomized animals in this study. All cell used in this study were randomly allocated into groups.
Blinding	For all experiments, the investigators were blinded to group allocation during data collection. The investigators were blinded when quantifying immunofluorescence. Fields for quantification were randomly selected and scored, as indicated in Methods.

Did the study involve field work? [x] Yes [ ] No

Field work, collection and transport

Field conditions	All experiments were performed in the labs at University of Rochester and Zhejiang University.
Location	All experiments were performed in the labs at University of Rochester and Zhejiang University.
Access & import/export	Tissues of Transcaucasian mole vole (TMV, Ellobius lutescens) were obtained from Dr. Yuksel Coskun lab at Dicle University, Turkey. Star-nosed moles (SNM, Condylura cristata) were collected in New York State. Tissues and cells of eastern moles (EM, Scalopus aquaticus) and short-tailed shrews (shrew, Blarina brevicauda) were obtained from Dr. Richard Miller lab at University of Michigan. Collection of star-nosed moles was covered by License to Collect or Possess issued by New York State department of environmental conservation (Scientific #2850). For wild-collected animals, three to five young adults with random sexes were obtained unless indicated otherwise in the Figure legends.
Disturbance	Not applicable

Reporting for specific materials, systems and methods

We require information from authors about some types of materials, experimental systems and methods used in many studies. Here, indicate whether each material, system or method listed is relevant to your study. If you are not sure if a list item applies to your research, read the appropriate section before selecting a response.

Materials & experimental systems

n/a	Involved in the study
[x]	[ ] Antibodies
[ ]	[x] Eukaryotic cell lines
[x]	[ ] Palaeontology and archaeology
[ ]	[x] Animals and other organisms
[x]	[ ] Clinical data
[x]	[ ] Dual use research of concern
[x]	[ ] Plants

Methods

n/a	Involved in the study
[x]	[ ] ChIP-seq
[x]	[ ] Flow cytometry
[x]	[ ] MRI-based neuroimaging

Eukaryotic cell lines

Policy information about cell lines and Sex and Gender in Research

Cell line source(s)	Primary fibroblasts were isolated from lung and skin of BMR, NMR, DMR, EM, SNM, Shrew, GP, and mice of at least three individuals. 293T and HeLa cells were obtained from ATCC.
Authentication	Fibroblast cell lines were authenticated using Short Tandem Repeat (STR) analysis as described in 2012 in ANSI Standard (ASN-0002) by the ATCC Standards Development Organization (SDO) and in Capes-Davis et al., Match criteria for human cell line authentication: Where do we draw the line? Int J Cancer. 2013;132(11): 2510-9. 293T and HeLa cells were obtained from ATCC and used at low passage. They were not further authenticated.
Mycoplasma contamination	All cells were mycoplasma-free with regular checks performed by a LookOut Mycoplasma PCR (i.e.,polymerasechain reaction) Detection Kit (MP0035, Sigma-Aldrich).
Commonly misidentified lines (See ICLAC register)	None of these cell lines are listed in the International Cell Line Authentication Committee (ICLAC) database.

Animals and other research organisms

Policy information about studies involving animals; ARRIVE guidelines recommended for reporting animal research, and Sex and Gender in Research

Laboratory animals	Blind mole rats (BMR, Nannospalax galili), previously caught in Upper Galilee Mountains in Israel, were maintained in the colonies of

	University of Rochester. Naked mole rats (NMR, Heterocephalus glaber) and Damaraland mole rats (DMR, Fukomys damarensis) were from the colonies at University of Rochester.
Wild animals	Tissues of Transcaucasian mole vole (TMV, Ellobius lutescens) were obtained from Dr. Yuksel Coskun lab at Dicle University, Turkey. Star-nosed moles (SNM, Condylura cristata) were caught in New York State. Tissues and cells of eastern moles (EM, Scalopus aquaticus) and short-tailed shrews (shrew, Blarina brevicauda) were obtained from Dr. Richard Miller lab at University of Michigan. Wild-collected star-nosed moles are covered by scientific license to collect or possess issued by New York State department of environmental conservation (Scientific #2850). For wild-collected animals, three to five young adults with random sexes were obtained unless indicated otherwise in the Figure legends.
Reporting on sex	The findings in this study do not have sex bias, and the sexes of animals were randomly obtained.
Field-collected samples	BMRs were housed in single cages at room temperature, with no light to mimic their subterranean habitat.
Ethics oversight	All animal experiments were approved by University Committee on Animal Resources of University of Rochester.

Note that full information on the approval of the study protocol must also be provided in the manuscript.
